# A COMPREHENSIVE EVALUATION OF END-OF-LIFE CARE EDUCATION NEEDS FOR HEALTH CARE PROFESSIONAL STUDENTS

**DOI:** 10.1093/geroni/igae098.0188

**Published:** 2024-12-31

**Authors:** Quynh Phung, Angelique King, Autumn Decker, Anna Roman, Logan Patterson, Raven Weaver, Cory Bolkan

**Affiliations:** Washington State University, Vancouver, Washington, United States; Washington State University, Spokane, Washington, United States; Pacific University, Hillsboro, Oregon, United States; Washington State University, Spokane, Washington, United States; Washington State University Elson S. Floyd College of Medicine, Spokane, Washington, United States; Washington State University, Pullman, Washington, United States; Washington State University, Pullman, Washington, United States

## Abstract

People are living longer than ever before, ushering in a need for more healthcare professionals to engage older patients about aging and end-of-life (EOL) values. Despite 99% of healthcare providers acknowledging the importance of advance care planning discussions, over two-thirds of physicians lack adequate training (Fulmer et al., 2018). To address this gap, rigorous evaluations of existing EOL education in professional health programs are needed. This study was conducted at a large public university’s Health Science campus; 84 healthcare students (i.e., medical, nursing, and “other” programs) completed a survey, exploring their educational and socio-emotional needs regarding current death education curricula. Findings revealed deficiencies in EOL education in professional training, with only 32% reporting any curricular exposure. Individuals with prior exposure to death exhibited less negative feelings about death and greater familiarity with EOL care topics (p< 0.05). Compared to other programs’ students, nursing students demonstrated a significantly higher overall familiarity of EOL topics (p< 0.05). However, there was still consistently lower familiarity in dementia directives and community resources across all programs. Over 83% expressed a strong desire for more death education. Though students favor comprehensive elective courses, time constraints make workshops and seminars more feasible. Important components in training include real exposure, reflection, and debriefs around EOL conversations or deaths. These findings underscore the urgent need for earlier and more comprehensive training in navigating complex EOL discussions and caring for older adults. Addressing these barriers has the potential to enhance professionals’ skills and wellbeing, improving quality of care provided for older adults.

